# Impact of continuous positive airway pressure on the treatment of acute exacerbation of asthma: a randomised controlled trial

**DOI:** 10.1186/cc12087

**Published:** 2013-03-19

**Authors:** Y Sutherasan, S Kiatboonsri, P Theerawit, C Kiatboonsri, S Trakulsrichai

**Affiliations:** 1 Ramathibothi Hospital, Bangkok, Thailand

## Introduction

Despite the extensive use of continuous positive airway pressure (CPAP) in various respiratory failure conditions, its role in acute asthmatic attack is uncertain [1-3]. This study aims at exploring the efficacy of CPAP when use in addition to the conventional treatment of acute asthma exacerbation in the emergency department (ED).

## Methods

During May to December 2009, acute asthma patients attending ED were randomly assigned to CPAP and control groups. In addition to the conventional treatment, CPAP ventilation of 8 cmH_2_O was applied to patients in the CPAP group. Data collections in both groups included peak expiratory flow rates (PEFR, %predicted) at each 15-minute interval (T0, T15, ...), the number of short-acting bronchodilators (SABA) used, ED length of stay, relapse rate and admission rate. The primary outcome is the improvement in peak expiratory flow rate during a short period stay in the ED.

## Results

There were 86 patients enrolled, with 43 patients in each study group. PEFR in the CPAP group showed no difference from the control group at T0 (52.7 ± 20.33 vs. 47.2 ± 16.64, *P *= NS) but was significantly better at T15 (65.58 ± 21.64 vs. 56.49 ± 18.53%, *P <*0.05). Such improvement was consistently observed for all subsequent PEFR measurement, along with the fewer SABA used and relapse rate, but the figures did not meet statistical difference.

## Conclusion

The addition of CPAP to conventional acute asthma treatment in the ED could accelerate PEFR improvement with a trend showing fewer SABA needed, lower relapse rate and shorter ED length of stay.
